# Properties of Basil and Lavender Essential Oils Adsorbed on the Surface of Hydroxyapatite

**DOI:** 10.3390/ma11050652

**Published:** 2018-04-24

**Authors:** Daniela Predoi, Andreea Groza, Simona Liliana Iconaru, Gabriel Predoi, Florica Barbuceanu, Regis Guegan, Mikael Stefan Motelica-Heino, Carmen Cimpeanu

**Affiliations:** 1 National Institute of Materials Physics, Atomistilor Street, No. 405A, P.O. Box MG 07, 077125 Magurele, Romania; simonaiconaru@gmail.com; 2 National Institute for Laser, Plasma and Radiation Physics, 409 Atomistilor Street, P.O. Box MG 36, 077125 Magurele, Romania; andreeagroza75@gmail.com; 3 Faculty of Veterinary Medicine, University of Agronomic Sciences and Veterinary Medicine of Bucharest, 105 Splaiul Independentei, Sector 5, 050097 Bucharest, Romania; gabrielpredoi@yahoo.com (G.P.); flori.barbuceanu@yahoo.com (F.B.); 4 Institute for Diagnosis and Animal Health, Bucharest, Romania, 63 Staicovici D. Nicolae, Street, 50557 Bucharest, Romania; 5 ISTO, UMR 7327 CNRS Université d’Orléans, 1A rue de la Férollerie, 45071 Orléans CEDEX 2, France; regis.guegan@univ-orleans.fr (R.G.); mikael.motelica@univ-orleans.fr (M.S.M.-H.); 6 Faculty of Science and Engineering, Global Center for Science and Engineering, Waseda University, 3-4-1, Okubo, Shinjuku-ku, Tokyo 169-8555, Japan; 7 Faculty of Land Reclamation and Environmental Engineering, University of Agronomic Sciences and Veterinary Medicine of Bucharest, 59 Marasti Blvd, Sector 1, 011464 Bucharest, Romania; carmencimpeanu@yahoo.com

**Keywords:** hydroxyapatite, plant essential oil, Gram positive bacteria, Gram negative bacteria

## Abstract

The research conducted in this study presented for the first time results of physico-chemical properties and in vitro antimicrobial activity of hydroxyapatite plant essential oil against Gram-positive bacteria (methicillin-resistant *Staphylococcus aureus* (MRSA) and *S. aureus* 0364) and Gram-negative bacteria (*Escherichia coli* ATCC 25922). The samples were studied by scanning electron microscopy (SEM) and Fourier transform infrared (FTIR) spectroscopy to determine the morphology and structure of the nanocomposites of hydroxyapatite coated with basil (HAp-B) and lavender (HAp-L) essential oils (EOs). The values of the BET specific surface area (S_BET_), total pore volume (V_P_) and pore size (D_P_) were determined. The results for the physico-chemical properties of HAp-L and HAp-B revealed that lavender EOs were well adsorbed on the surface of hydroxyapatite, whereas basil EOs showed a poor adsorption on the surface of hydroxyapatite. We found that the lavender EOs hydroxyapatite (HAp-L) exhibited a very good inhibitory growth activity. The value of the minimum inhibitory concentration (MIC) related to growth bacteria was 0.039 mg/mL for MRSA, 0.02 mg/mL for *S. aureus* and 0.039 mg/mL *E. coli* ATCC 25922. The basil EO hydroxyapatite (HAp-B) showed poor inhibition of bacterial cell growth. The MIC value was 0.625 mg/mL for the HAp-B sample in the presence of the MRSA bacteria, 0.313 mg/mL in the presence of *S. aureus* and 0.078 mg/mL for *E. coli* ATCC 25922.

## 1. Introduction

Hydroxyapatite (HAp) is a calcium phosphate compound and the best known biomaterial in terms of its morphological and compositional similarity with human hard tissue. From a thermodynamic point of view, hydroxyapatite is the most stable calcium phosphate with regard to the physiological conditions of the human body, including temperature, body fluids composition and human pH. The outstanding properties of HAp nanopowders, such as their biocompatibility, bioactivity, osteoconductivity and nontoxicity, have been demonstrated [1].

Hydroxyapatite inorganic components (Ca_10_(PO_4_)_6_(OH)_2_) resemble, in the most favorable way, the natural biological apatite found in bone. Therefore, it is of interest in medicine and biomedical engineering for bone regeneration and dental applications. As a result of its biocompatibility, hydroxyapatite can be used for coating metal prostheses or dental implants, which is favorable for a successful osteointegration process. On the other hand, it is well known that the risk of postoperative infections [2,3,4] can be drastically reduced by incorporating antibacterial agents in the chemical structure of hydroxyapatite.

Essential oils are complex biostructures, and contain a lot of chemical compounds from different chemical classes: terpenoids, ketones, aldehydes, and esters, either saturated or with different levels of unsaturation, their chemical composition depending on the plant’s origin and quality, harvest time, climate, soil and especially extraction process [3,4,5,6,7]. About 90% of the bioactive components of EOs are monoterpenes. In oxygenated form, the chemical constituents of EOs exhibit a better bioactivity.

Essential oils have been used since ancient times. Over the years, EOs have been approved for use in cosmetics, pharmaceuticals, perfumery and food and medicine industries, and are some of the most commonly used natural products. On the other hand, aromatherapy, a branch of alternative medicine, argues that essential oils can be particularly active healing remedies with specific actions for various maladies. Moreover, they can be a natural alternative to synthetic drugs, which produce countless side effects. Today, by combining science with ancient wisdom, we can gain a deeper understanding of and, at the same time, access to the therapeutic benefits that essential oils possess [8]. The essential oils of basil and lavender are among the best known because of their applicability in various fields of activity. Lavender EOs contains volatile molecules of particular interest to the cosmetics industry, perfumes and aromatherapy, while basil EOs are commonly used in the food industry.

In recent years, because of the excessive use of antibiotics, bacteria and viruses are becoming more resistant to drugs. On the other hand, viruses and bacteria have changed a lot over time, and antibiotics cannot treat most of the diseases they cause. Even if bacteria adapt and become resistant to antibiotics, medical and pharmaceutical specialists are trying to find new products that can be used to develop medicines to fight back against pathogens. More than that, chemically synthetized drugs present a certain toxicity and involve a potential risk to humans. In this context, researchers are continuously trying to find new molecules to fight against bacterial strains.

Recent studies in this field [8] have shown that essential oils are encouraging for use in medicine and biomedical applications due to their antibacterial, antifungal and antiviral properties and their ability to prevent the growth of different pathogens. Lavender oil chemical components were proved to be inhibitors of *M. smegmatis* and *E. coli* microorganisms [8]. Basil oils (*Ocimum basilicum*) exhibit antibacterial activity against *Brochothrix thermosphacta*, *E. coli*, *L. innocua*, *L. monocytogenes*, *P. putida*, *S. typhimurium*, *S. putrefaciens* and *M. flavus* microorganisms [8].

In this context, the results presented in this paper evidence the physicochemical properties and antibacterial activities of hydroxyapatite-lavender essential oil (HAp-L) and hydroxyapatite-basil essential oil (HAp-B) nanocomposites. The morphology of the surfaces of the hydroxyapatite nanoparticles coated with essential oils was analyzed by Scanning Electron Microscopy (SEM). The influence of the basil and lavender essential oils on the molecular structure of the HAp was investigated using Fourier Transform Infrared Spectroscopy measurements. The antibacterial effect of hydroxyapatite coated with basil (HAp-B) and lavender (HAp-L) essential oil nanopowders (pressed into pellets) against Gram-positive (MRSA, *S. aureus*) and Gram-negative (*E. coli*) bacteria was also studied.

## 2. Materials and Methods

The plant EOs used in this study were lavender oil, naturally obtained from *Lavandula angustifolia* L. (Sigma Aldrich, St. Louis, MO, USA), and basil oil, linalol type, obtained from *Ocimum basilicum* L. (Sigma Aldrich). The essential oils were used as purchased without any dilution.

The Ca_10_(PO_4_)_6_(OH)_2_ hydroxyapatite powders (HAp) were synthesized by an adapted coprecipitation method maintaining the molar ratio Ca:P = 1:67 [9] and using as precursors calcium nitrate (Ca(NO_3_)_2_∙4H_2_O, Sigma Aldrich, St. Louis, MO, USA), ammonium hydrogen phosphate ((NH_4_)_2_HPO_4_; Wako Pure Chemical Industries Ltd., Richmond, VA, USA), ammonium hydroxide (NH_4_OH, Wako Pure Chemical Industries Ltd., Richmond, VA, USA), and double distilled water. Ca(NO_3_)_2_·4H_2_O and (NH_4_)_2_HPO_4_ were dissolved in deionized water and stirred vigorously for 2 h. The solution containing P was then added dropwise into the Ca-containing solution, and the new obtained mixture was magnetically stirred for 2 h at a temperature of 100 °C. During the reaction, the pH value was constantly kept at 10. The final product was washed with deionized water several times. The obtained material (HAp) was dried for 48 h at 100 °C. The HAp-B and HAp-L samples were obtained by mixing HAp powder with basil and lavender essential oil. HAp, HAp-B and HAp-L powders were pressed into pellets with a diameter of 6 mm.

The morphology of the samples was investigated by Scanning Electron Microscopy (SEM) using a HITACHI S4500 microscope (Hitachi, Ltd., Tokyo, Japan). Prior to recording the images, the powder samples were evenly dispersed in a specimen holder using as double coated conductive carbon tape conductive adhesive. The sample holder containing the samples was introduced into the vacuum chamber, and all SEM images were recorded using a 5 kV electron acceleration voltage and an Everhart-Thornley detector (ETD). The 3D surface plots of the SEM images were obtained using Image J software (ImageJ 1.51j8, National Institutes of Health, Bethesda, MD, USA) [10].

The specific surface area (S_BET_) cumulative pore volume (V_p_) and diameter of the pores (D_p_) of the HAp, HAp-B, HAp-L samples were determined using an ASAP 2020 instrument from the nitrogen adsorption-desorption isotherm. The measurements were performed at 77 K. The specific surface areas of the HAp, HAp-B, and HAp-L samples were determined by Brunauer-Emmett-Teller (BET) method [11]. The total pore volume (V_P_) and pore size (D_P_) were calculated using the Barrett, Joyner, and Halenda (BJH) method [12].

The spectra of HAp-B and HAp-L samples were acquired using a Perkin Elmer spectrometer (Waltham, MS, USA) SP 100 equipped with an Attenuated Total Reflection (ATR) accessory, in the spectral range of 400–4000 cm^−1^ with a 4 cm^−1^ resolution. The samples were placed on the ATR accessory without any preparation. The background spectrum was acquired in air and taken as reference background spectrum. Before and after the spectral analysis of each sample, the ATR diamond crystal was cleaned with an isopropyl alcohol solution. The ATR plate cleaning was verified by recording the background spectrum after each sample measurement and comparing it with the reference background spectrum. The acquired transmission IR spectra were transformed into absorption spectra according to the A = log (1/T) using the Perkin Elmer spectrometer, SPECTRUM software (Version 6.4.1, Perkin Elmer, Waltham, MS, USA). The fine structure of the obtained absorption spectra of the HAp, HAp-B, and HAp-L samples was revealed by peak fitting analysis. The first step performed in the peak fitting analysis procedure was the baseline correction of the experimental infrared spectrum, followed by the second-order derivative calculation for peak wavenumbers finding. Secondly, during the fitting analysis process, a Lorentz-type profile curve was used for each identified peak. A sum of all the Lorentz fitted curves was generated by multiple iteration of the nonlinear least-squares data-fitting algorithm of the calculation software. If the sum of all the fitted curves is set correctly, the algorithm gives a valid and convergent solution. The theoretical curve obtained as a result of peak fitting analysis versus experimental IR curve was presented and analysed [13,14]. 

The tested microorganism strains that were used in this study were Gram-positive bacteria methicillin-resistant *Staphylococcus aureus* (MRSA) and *Staphylococcus aureus* 0364 (*S. aureus* 0364) and Gram-negative bacteria *Escherichia coli* ATCC 25922, (*E. coli* ATCC 25922). The qualitative antibacterial assays were performed using microbial suspensions obtained from 15–18 h solid cultures on tryptone soy agar (TSA). The cultures were then transferred to fresh Mueller-Hinton agar (MHA) and incubated for 2–3 h to reach the exponential growth phase. The bacterial suspensions were adjusted to 0.5 McFarland standard (1 × 10^8^ CFU/mL) and were spread on Mueller-Hinton agar plates evenly using a sterile swab. Hydroxyapatite, and hydroxyapatite coated with lavender and basil EOs pellets were placed directly, aseptically and distinctively onto the previously inoculated MHA plates. Agar plates were incubated at 37 °C for 18–24 h and the inhibition zones formed were measured in mm [15,16]. The quantitative studies regarding the antibacterial activity of the HAp, HAp-B and HAp-L powders were performed by microdilution broth method in 96 multi-well plates [17,18,19]. The materials were solubilized in DMSO and two-fold dilutions of the samples ranging from 5 to 0.01 mg/mL were carried out in Mueller-Hinton broth (MHB). Each tube was inoculated with microbial suspension adjusted to 0.5 McFarland and a volume of 100 μL of each sample dissolved in DMSO as described above were added to the first well and a serial two-fold dilution was performed. After incubation at 37 °C for 18–24 h, the minimum inhibitory concentration (MIC) values and minimum bactericidal concentration (MBC) values were determined by measuring the absorbance of the microbial suspensions at 620 nm.

## 3. Results and Discussion

The microstructure of the HAp sample and the influence of the basil and lavender EOs on the hydroxyapatite nanoparticles’ shape and distribution were investigated by SEM.

**Figure 1:**
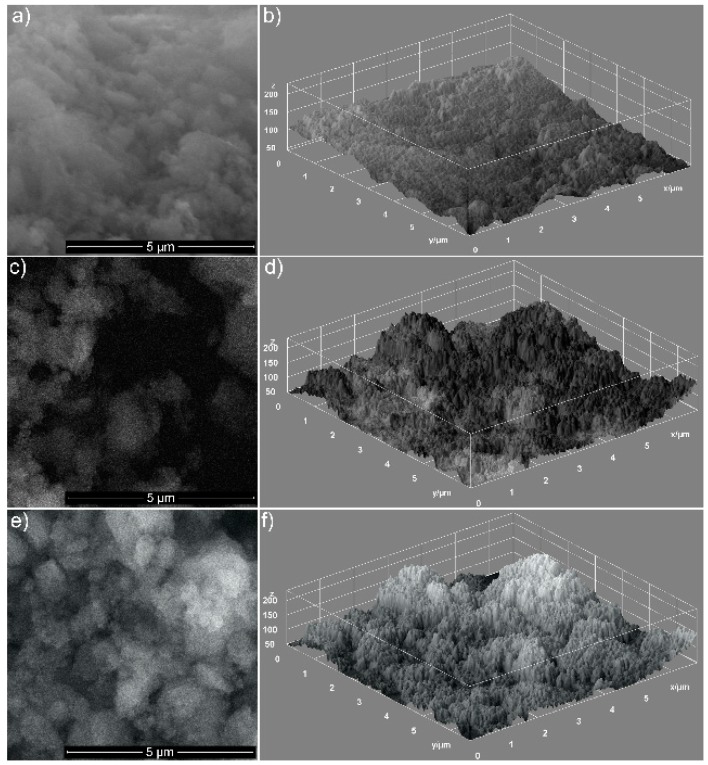
The SEM images of HAp (**a**), HAp-B (**c**) and HAp-L (**e**). 3D surface plot of SEM images of HAp (**b**), HAp-B (**d**) and HAp-L (**f**) samples.

The respective morphologies of the hydroxyapatite coated with EOs of basil (HAp-B) and lavender (HAp-L) are shown in Figure 1a–c. In Figure 1a, the uniform ellipsoidal shape of HAp nanoparticles and their distribution can be observed in the investigated sample. By covering HAp with EOs, the nanoparticles agglomerated, as can be observed in Figure 1b,c. The particles’ shape remained ellipsoidal for both HAp-B and HAp-L samples, while the sample surfaces were affected.

Information on the uniformity and the homogeneity of the analyzed samples is provided in Figure 1b,d,f. The SEM images are shown as 3D surface plots (Figure 1b,d,f) of HAp, HAp-B and HAp-L samples obtained using Image J software (ImageJ 1.51j8, National Institutes of Health, Bethesda, MD, USA) [10] are also presented. The distribution of the HAp nanoparticles covered with EOs became non-uniform, and it seemed that the porosity of the HAp nanoparticles increased. An obvious increase in porosity was noticed for HAp-L samples, while a clear change in the porosity of HAp-B samples was not observed. This behavior could suggest that basil EO had poor adsorption on the surface of hydroxyapatite nanoparticles.

The particle size, structure, and morphology can have a significant influence on both materials and the efficiency of their use. Properties such as S_BET_, V_P_ and D_P_ are significant material characteristics in numerous applications, including pharmaceutical and medical products. To determine the total pore volume (V_P_) and pore size (D_P_) of the HAp, HAp-B, HAp-L samples, the Barrett, Joyner, and Halenda (BJH) method was used. Moreover, the values of the specific surface area (S_BET_) of the samples were determined by the BET method. The nitrogen physisorption results, including the S_BET_, V_P_ and D_P_ of the HAp, HAp-B, HAp-L samples, are given in Table 1.

The BET surface areas increased from HAp to HAp-L. The S_BET_ value for HAp-B is close to that of HAp. As expected, the pore volume V_P_ increased from HAp to HAp-L. The results regarding the porosity of the materials were similar to those observed by SEM analysis. These materials could have high potential for use in a variety of applications, due to the possibility of obtaining particles with a controlled size and shape. 

**Figure 2:**
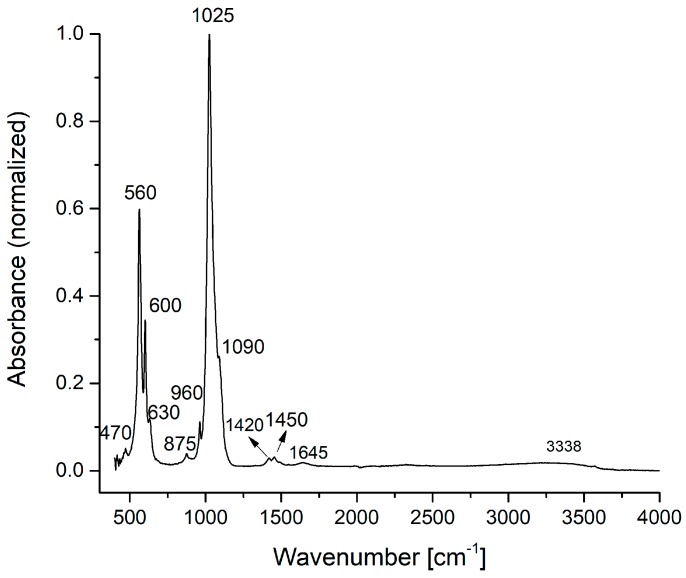
FTIR spectra of the HAp sample.

**Figure 3:**
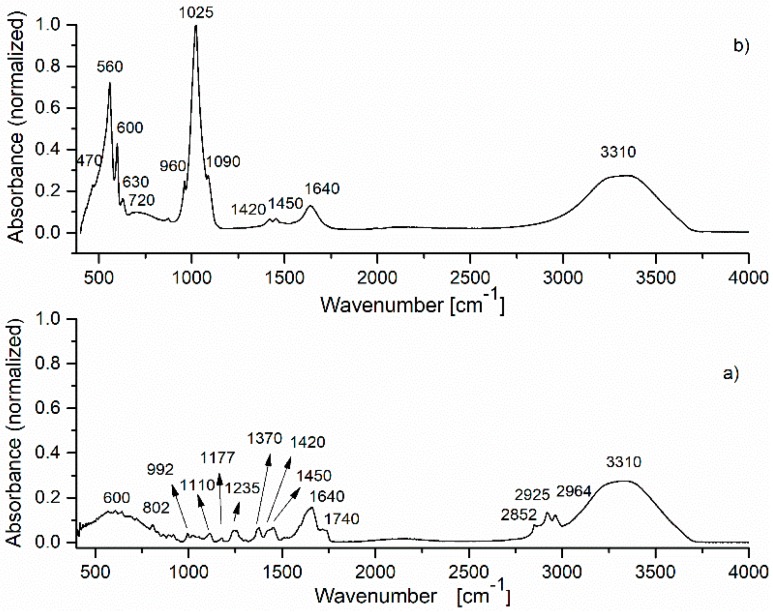
FTIR spectrum of: (**a**) basil essential oil; (**b**) HAp-B sample.

**Figure 4:**
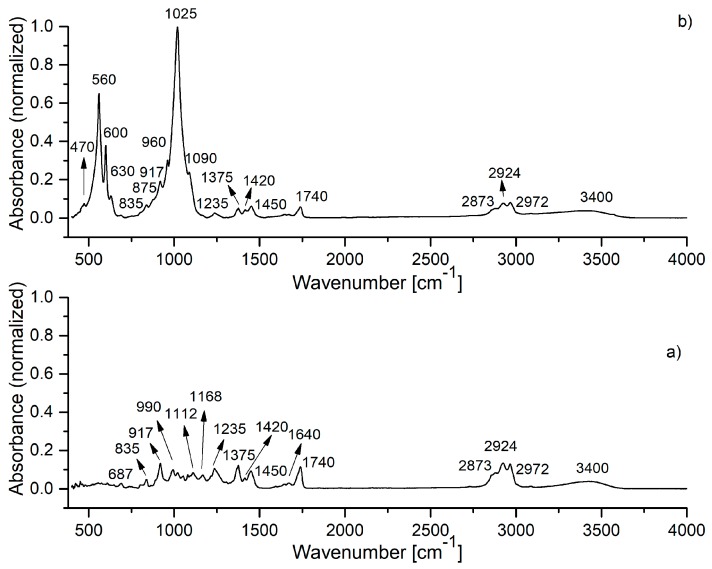
FTIR spectrum of: (**a**) lavender EO; (**b**) HAp-L sample.

The IR spectra of HAp, HAp-B and HAp-L samples and IR band assignments for HAp, HAp-B and HAp-L are presented in Figure 2, Figure 3 and Figure 4 and Table 2.

In the IR absorption spectrum from Figure 2, the main characteristic vibrational bands of the HAp nanoparticles can be identified. The spectrum was normalized from 0 to 1 using the SPECTRUM software of the Perkin Elmer SP 100 spectrometer. The fundamental vibrational modes of [PO_4_]^3−^ groups of the apatitic structure appear at 470 cm^−1^ (ν_1_), 560, 600, 630 cm^−1^ (ν_4_), 960 cm^−1^ (ν_1_), 1025, 1090 cm^−1^ (ν_3_) [20,21]. The IR bands from 875, 1420, 1450 cm^−1^ can be attributed to the vibrations of the [CO_3_]^2−^ carbonate group [20]. The [CO_3_]^2−^ group absorption band presence in the HAp IR spectrum was due to the formation of carbonated groups in the HAp powder during the chemical synthesis. The weak and broad IR bands from 1645 and 3338 cm^−1^ characterize the O–H vibration from the water absorbed into the HAp structure.

The IR spectra of the basil EO and HAp-B samples are presented in Figure 3. According to Figure 3a [6,18], in the spectrum of basil EO, the main characteristic IR absorption bands were observed. In the 2800–3400 cm^−1^ spectral region, the IR peaks of –CH_2_ stretching (2852, 2925, 2964 cm^−1^) and C–H stretching (3310 cm^−1^) vibrations were identified. The intense IR band at 1640 cm^−1^ was attributed to the C=C stretching vibration and the 1740 cm^−1^ absorption band is characteristic of C=O stretching vibrations. The most relevant absorption bands from the 500–1500 cm^−1^ spectral region are: 600, 802 (C–H vibrations), 1110, 1177, 1235 cm^−1^ (C–O stretching vibrations), 992, 1370, 1420, 1450 cm^−1^ (C–H bending vibrations) [22]. 

The spectrum of the HAp-B sample was dominated by the IR bands specific to HAp. The IR spectral absorption bands of basil EO were usually present in the 2700–3200 cm^−1^ and 600–1850 cm^−1^ spectral regions. As the 600–1850 cm^−1^ spectral range was mainly dominated by the IR bands characteristic to HAp, only a few IR bands specific to basil oil were indicated in the HAp-B absorption spectrum shown in Figure 3 and subsequently in Table 2, like 720 cm^−1^ (C–H vibrations), 1640 cm^−1^ (O–H vibrations) respectively 3310 cm^−1^ (O–H vibrations). The broad IR band from 720 cm^−1^ observed in Figure 3a appears to be due to the overlapping of the basil EO absorption bands in the 600–800 cm^−1^ spectral region.

Furthermore, the normalized absorption IR spectra of basil EO and HAp-B presented in Figure 3 could provide a quantitative evaluation of the basil EO absorption into the HAp sample. By comparing the IR spectrum of HAp (Figure 2) with the IR spectrum of HAp-B (Figure 3b), wavenumber differences can be noticed, especially in the 1400–4000 cm^−1^ range. On the other hand, in the IR spectrum of the HAp-B sample, the bands specific to basil EO were not clearly highlighted, although there are many IR-specific bands of basil EO in the 400–1400 cm^−1^ region. This is also the case concerning the IR bands at 2852, 2925, and 2964 cm^−1^. In the spectrum of the HAp-B sample, only the basil EO IR absorption bands of 1640 and 3310 cm^−1^, attributed to O-H vibrations, were visible (Figure 2). These bands were partially overlaid with the 1645 and 3338 cm^−1^ IR bands characteristic to HAp (Figure 2). These results could indicate a poor absorption of basil EO into the HAp structure. The absorption intensities of the 1640 and 3310 cm^−1^ IR bands in the basil EO spectrum were 0.15 and 0.26, respectively. In the HAp-B spectrum, the intensities of these absorption bands were 0.13 and 0.27, respectively.

The IR spectra of lavender EO and HAp-L samples are presented in Figure 4. The IR spectrum in Figure 4a reveals the characteristic IR absorption bands of lavender EO [6,18]. In the spectral region of 2800–3400 cm^−1^, the IR peaks at 2873, 2924, 2972 cm^−1^ were ascribed to –CH_2_ stretching, while the band at 3400 cm^−1^ was attributed to O–H stretching vibrations. The most intense IR band in the spectrum appears at 1740 cm^−1^ (C=O stretching vibrations). The 1640 cm^−1^ IR band belongs to the C=C stretching vibration. In the 400–1500 cm^−1^ region, the following IR peaks were identified: 687, 835, 917 cm^−1^ (C–H vibrations), 990 cm^−1^ (–CH_2_ vibrations), 1112, 1168, 1235 cm^−1^ (C–O stretching vibrations), 1375, 1420, 1450 cm^−1^ (C–H bending vibrations) [22]. 

In Figure 4b, the IR spectrum of the HAp-L samples reveals absorption bands characteristic to both HAp and lavender EO. All the characteristic IR absorption bands for HAp were present in the spectrum. In addition to the HAp IR spectral bands, the IR bands specific to lavender EO can also be observed in Figure 4b. Thus, the bands at 692, 917, 1375 cm^−1^ correspond to C–H vibrations, while the band at 1640 cm^−1^ was associated with the C=C stretching vibrations [23,24]. The 1235 cm^−1^ IR band belongs to the C–O stretching vibration of the ester group, and the 1740 cm^−1^ band was related to the C=O stretching vibration. The IR bands from 1375 cm^−1^ and those in the 2800–3000 cm^−1^ range were due to the bending and stretching of the C–H vibrations in methyl and methylene groups, as was indicated in Table 2. The broad band at 3400 cm^−1^ (O–H) indicated an alcohol presence [23,24].

The HAp-L IR spectrum from Figure 4b reveals the IR bands of lavender EO even in the 400–1500 cm^−1^ spectral region, which is dominated mainly by the HAp IR bands. In the wavenumber range 1500–4000 cm^−1^, the lavender EO IR bands at 1740, 2873, 2924, 2972 and 3400 cm^−1^ specific to C=O stretching, methylene stretching and O–H vibrations can be clearly identified. The values of the absorption intensities of the IR bands characteristic to lavender EO in both lavender EO and HAp-L IR spectra, as measured from the IR spectra in Figure 4 in the 1200–4000 cm^−1^ spectral region, are presented in Table 2. The absorption bands at 1235, 1375, 1740, 2873, 2924, 2972 cm^−1^ are the only ones that are not overlaid with the HAp IR bands. Comparison of the intensities of these absorption bands in both spectra from Figure 4 can give a quantitative estimation of the degree of the lavender EO absorption into the HAp structure. Moreover, the spectra from Figure 4 and the data presented in Table 3 indicate better absorption of lavender EO into the HAp structure in comparison with the basil EO.

According to previous studies [23,24] regarding the IR analysis of essential oils such as lavender and basil essential oils, there are several absorption bands in the 800–1200 cm^−1^ range. In the IR spectrum of the HAp-B and HAp-L samples, only some of these were observed. The same comportment was observed in the case of the HAp sample.

Thus, in order to discover the complete molecular structural information of the HAp, HAp-B and HAp-L samples, we performed a peak fitting analysis of their IR spectra in the 800–1200 cm^−1^ spectral range. As a first step in the peak fitting analysis procedure, the second derivative of the IR spectrum was calculated.

**Figure 5:**
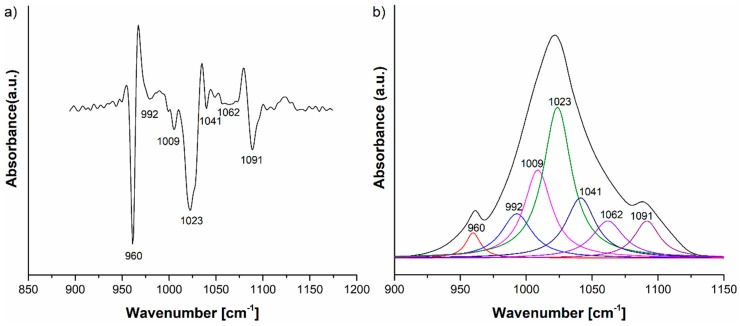
(**a**) Second derivative and (**b**) FTIR deconvoluted spectra of the HAp-B IR spectrum in the 800–1200 cm^−1^ domain.

The HAp-B IR deconvoluted spectrum is presented in Figure 5, together with the second derivative curve in the 800–1200 cm^−1^ spectral range. The 960, 1009, 1023, 1041, 1062 and 1091 cm^−1^ IR absorption bands can be attributed to the P-O vibrations in the [PO_4_]^3−^ group, while the IR band at 992 cm^−1^ can be assigned to the C–H vibrational deformations present in basil EO [23,24]. 

**Figure 6:**
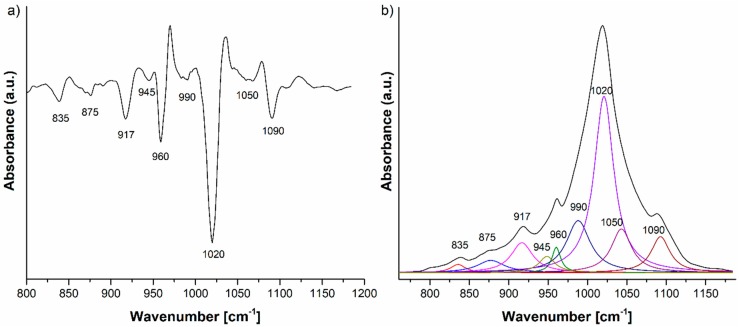
(**a**) Second derivative and (**b**) FTIR deconvoluted spectrum of the HAp-L IR spectrum in the 800–1200 cm^−1^ domain.

In the case of the HAp-L IR deconvoluted spectrum (Figure 6b), the peaks from 835, 875 cm^−1^ are attributed to the C–O vibrations in the [CO_3_]^2−^ group, an impurity in the synthesis of the HAp. The peak at 835 cm^−1^ could also be assigned to the C–H vibrations into the lavender EO spectrum (Figure 4). The 920, 945, 960, 1020, 1050 and 1090 cm^−1^ IR peaks belong to the P–O vibrations in the [PO_4_]^3−^ group [20,21]. In addition to the peaks previously identified in the IR spectrum of HAp-L, the peak fitting analysis revealed some peaks related to the structure of the lavender EO, namely 917 and 990 cm^−1^, assigned to C-H vibrational deformations in accordance with [23,24].

The second derivative and, consequently, the deconvoluted IR spectra of the HAp-B, HAp-L and HAp samples in the 800–1200 cm^−1^ spectral region indicate slight differences in the wavenumbers of the IR peaks belonging to the P–O vibrations (Figure 5a and Figure 6a). The ν_3_ fundamental vibrational mode of [PO_4_]^3−^ group is usually formed in the 1000–1100 cm^−1^ range [25], so this implies that some interactions take place between the atoms and molecules of the HAp and basil EO structure and the HAp and lavender EO structure.

The IR spectrum of the HAp-B sample in Figure 3b, as well as the second derivative and the deconvoluted IR spectra from Figure 5, indicate only a few IR bands specific to basil EO in comparison with the bands specific to lavender EO. In [23,26], it was shown that the basil and lavender EOs have many characteristic IR bands in the 800–1800 cm^−1^ spectral region. In the HAp-B IR spectrum, there were a few IR bands specific to the basil EO (Table 2, Figure 3 and Figure 5). In the IR spectrum of HAp-L, more IR bands specific to lavender EO were identified (Table 2, Figure 4 and Figure 6). This emphasizes the fact that the lavender EO was better adsorbed on the surface of hydroxyapatite compared to basil EO.

**Figure 7:**
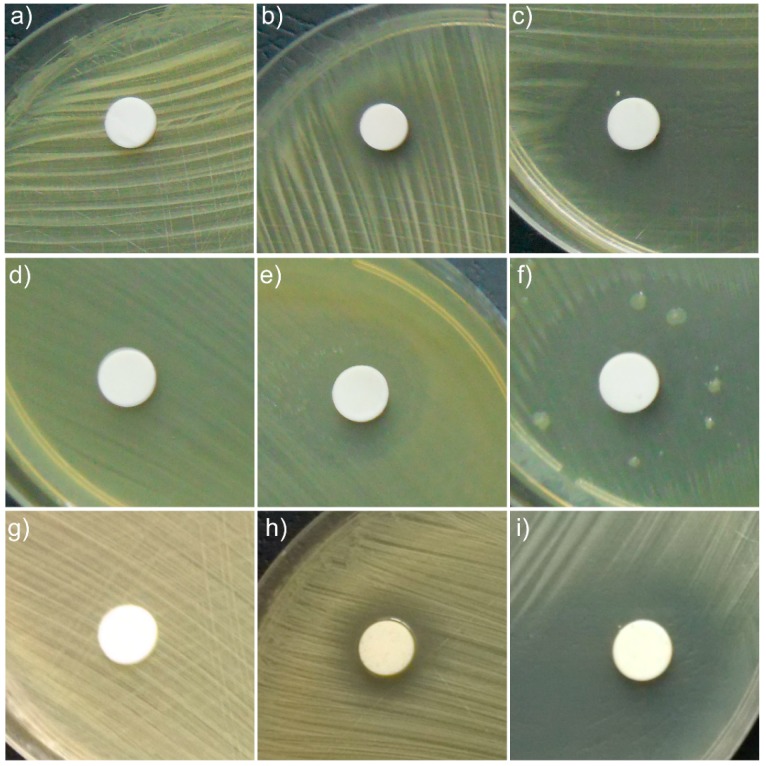
Qualitative assay of the inhibitory activity of HAp, HAp-B and HAp-L samples against MRSA (**a**–**c**), *E. coli* ATCC 25922 (**d**–**f**) and *S. aureus* 0364 (**g**–**i**) bacterial strains.

The antibacterial activity of the HAp samples and HAp coated with essential oil of basil and lavender samples was assessed using Methicillin-Resistant Staphylococcus aureus (MRSA) and *Staphylococcus aureus* 0364 (*S. aureus* 0364) and Gram-negative bacteria, *Escherichia coli* ATCC 25922, (*E. coli* ATCC 25922) bacterial strains. The qualitative antibacterial results of the tested samples obtained by an adapted disc diffusion method are illustrated in Figure 7, and the inhibition zone diameters measured in mm are presented in Table 4.

The results of the antibacterial assays emphasized that HAp pellet powders exhibited no antimicrobial activity against the tested Gram-positive and Gram-negative bacterial strains.

The results indicated that HAp-L displayed good antibacterial activity against MRSA, *S. aureus* 0364 and *E. coli* ATCC 25922 microbial strains, while the antibacterial activity of HAp-B samples against MRSA, *S. aureus* 0364 and *E. coli* ATCC 25922 was not so well evidenced. It can be observed that HAp-L was strongly inhibitory against all tested bacterial strains (Figure 7c,f,i). The results also suggested that HAp-B was moderately inhibitory against *S. aureus* 0364 (Figure 7h) and weakly inhibitory to MRSA, (Figure 7b). The HAp-B sample showed the best antimicrobial activity against the *E. coli* ATCC 25922 bacterial strain (Figure 7e). Due to the fact that the HAp powders presented no inhibitory effect on the growth of the tested bacterial strains, the antibacterial activity of the HAp-B and HAp-L samples was attributed to the basil and lavender EOs. According to the manufacturer’s data, the basil essential oil (W212000 Aldrich) used in this study was mainly composed of eugenol, 32.4%; limonene, <0.1%; linalool, 41.9%; and methyl chavicol, 0.9% [27]. The lavender essential oil (61718 Aldrich) was comprised of over 100 constituents, including linalool, perillyl alcohol, linalyl acetate, camphor, limonene, tannins, triterpenes, coumarins 3,4,5, cineole, and flavonoids, etc. [28,29]. Both essential oils, purchased from Sigma Aldrich, had as a major constituent linalool, which has been reported to possess a strong antimicrobial activity [30]. Studies regarding the synergy between the components of essentials oils are still scarce. However, numerous results in this area have reported that the antimicrobial activity of a given essential oil is strongly influenced by just one or two main components, and also by the interactions between the major and minor components present in the oil composition [31]. Therefore, the distinctive behavior of the antimicrobial activity of HAp-B and HAp-L samples attributed to the basil and lavender EOs could be related both to the different interactions between the two oils constituents with the HAp powders and to the synergy between the constituent elements of each oil, separately. The effectiveness of essential oils in inhibiting bacterial growth is strongly depended on the nature of functional groups and orientation. Various mechanisms explaining the antimicrobial activity of EOs have been reported in the literature. EOs can disrupt the cell membrane of a microorganism by simply increasing membrane permeability, destabilizing the cellular architecture causing the breakdown of membrane integrity, which disrupts many vital cellular activities [32]. Therefore, the antimicrobial activity of EOs differs depending on the type of EO, as well as the targeted microbial strain, depending on their structure (Gram positive, Gram negative and fungi). It has been reported that sandalwood and vetiver EOs have strong inhibitory effects on the Gram-positive bacterial strains, while they do not exhibit any antibacterial activity against Gram-negative bacterial strains [33,34]. On the other hand, EOs obtained from cinnamon, clove, pimento, thyme, oregano, and rosemary have been reported to be effective against *Salmonella typhi* (Gram negative), *Staphylococcus aureus* (Gram positive), and *Pseudomonas aeruginosa* (Gram negative) bacterial strains [35]. Moreover, *Myrtus communis* EO has been described as being effective in the case of *S. aureus*, *L. monocytogenes*, *Enterococcus durans*, *B. subtilis, Mycobacterium tuberculosis*, *P. aeruginosa*, *S. typhi*, *E. coli*, *K. pneumoniae*, and *Mycobacterium avium* bacterial strains, which belong both to the Gram-negative as well as the Gram-positive bacterial strains [36,37]. Even though there are numerous studies regarding the antibacterial activity of EOs, there is no substantial evidence that definitely supports EOs presenting stronger antimicrobial activity against Gram-positive bacteria compared to Gram-negative bacteria. The different behavior of HAp-B and HAp-L samples could also be due to different adsorption on the hydroxyapatite surface of the two EOs (basil and lavender) as can be seen in the studies presented above.

These findings are in good agreement with studies regarding the antibacterial activity of lavender EO, which was demonstrated to exhibit in vitro activity against MRSA (methicillin-resistant *Staphylococcus aureus*) even at low concentrations [38,39,40,41,42,43,44]. In recent studies on the antimicrobial activity of EOs from plants against selected pathogenic and saprophytic microorganisms [44], it was concluded that essential oils extracted from oregano, basil, and coriander plants exhibit a noticeable inhibitory effect against *P. aeruginosa*, *S. aureus*, and *Yersinia enterocolitica* even at low concentrations. On the other hand, M. Sienkiewicz et al. [41], following studies on the use of essential oils of rosemary as effective antibacterial agents, reported that basil EO exhibited strong antibacterial activity against *E. coli* clinical strains.

The antibiotic resistances of the tested microorganisms have been investigated and reported in numerous studies [41,42,43,44,45,46]. In their research, A.W. Khan et al. [42] identified a zone with an average inhibition diameter of 23 mm for *E. coli* ATCC 25922 when tested against antibiotic discs of tetracycline (30 µg). More than that, the antibiotic resistance of different types of MRSA has been investigated by Trzcinsky et al. [43]. They showed that the diameter of the inhibition zone is closely related to clinical isolation, reporting an area with an average inhibition diameter of 6 to 17 mm when the antibiotic resistance of different types of MRSA was tested with tetracycline antibiotic discs (30 μg). The antibiotic resistance of *S. aureus* control strains against antibiotic discs of tetracycline (30 µg) has also been tested previously [44,45], indicating that the diameter of the inhibition zone was between 19 and 28 mm.

The antibacterial activity of the HAp-B and HAp-L samples was also confirmed by measuring the inhibition zone diameters (Table 4). The antimicrobial activity of HAp. HAp-B and HAp-L ranged from no inhibition for HAp sample to 25.83 ± 0.9 mm for the HAp-L sample in the case of *S. aureus* 0364 bacterial strain. The results suggested that HAp-L presented a stronger antibacterial activity against all tested microbial strains. In the case of HAp-B sample, the *E. coli* bacterial strain proved to be the most susceptible, presenting a diameter inhibition zone of 14.65 ± 0.5 mm.

**Figure 8:**
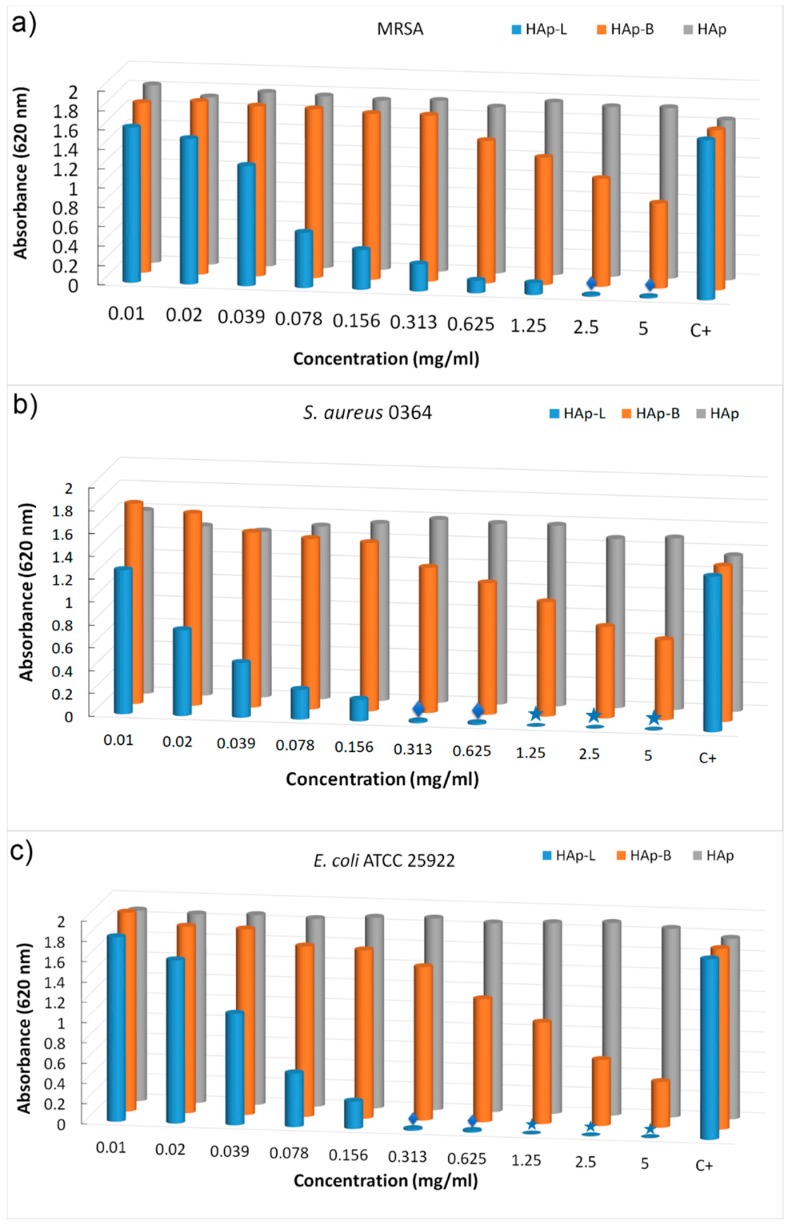
Graphic representation of absorbance values of the microbial culture obtained in the presence of the plant EO-coated HAp (HAp-B and HAp-L) on different bacterial strains such as MRSA (**a**), *S. aureus* (**b**) and *E. coli* (**c**), quantified by the A620 nm values.

The goal of this research was to obtain significant information about the antibacterial effect of nanopowders of hydroxyapatite coated with basil (HAp-B) and lavender (HAp-L) EOs against MRSA, *S. aureus* and *E. coli* bacterial strains (Figure 8).

The effect of nanopowders of HAp-B and HAp-L on cell viability was appraised on different Gram-positive (MRSA and *S. aureus*) and Gram-negative (*E. coli*) bacteria (Figure 8a–c). The effects of the HAp-B and HAp-L samples tested against MRSA cell growths at different concentrations from 0.01 to 5 mg/mL are presented in Figure 8a. For HAp-L the growth of MRSA cell was diminished at concentrations greater than 0.02 mg/mL. A slow decrease in MRSA cell growth was observed for HAp-B samples. HAp-B leads to a decrease in MRSA cell growth at concentrations greater than 0.625 mg/mL. It was noticed that at concentrations lower than 0.625 mg/mL, HAp-B did not have any effect on the inhibition of MRSA cell growth. An evolution of *S. aureus* cell growth in the presence of the HAp-B and HAp-L was also monitored (Figure 8b). Impaired cell growth of *S. aureus* was observed at concentrations between 0.313 and 5 mg/mL for the HAp-B sample. Likewise, a decrease in cell growth of *S. aureus* was observed, starting at the lowest concentration (0.01 mg/mL), in the presence of the HAp-B sample. Additionally, the inhibitory effect of the HAp-B and HAp-L was also evaluated against *E. coli* bacterial cells (Figure 8c). The HAp-L inhibited the *E. coli* growth starting with a concentration equal to 0.02 mg/mL while the HAp-B inhibited the *E. coli* growth starting with a concentration equal to 0.078 mg/mL.

Taking into account the fact that the MIC is the lowest concentration of a chemical that prevents visible growth of a bacterium, it can be said that the smallest concentration of HAp-L nanopowders at which the visible inhibition of MRSA bacterial growth was evidenced was 0.039 mg/mL. The MIC value for the HAp-B sample related to the MRSA was 0.625 mg/mL. On the other hand, the visible inhibition of *S. aureus* bacterial growth was observed at 0.02 mg/mL for the HAp-L sample. The value of MIC related to the growth of *S. aureus* bacteria was registered at 0.313 mg/mL for the HAp-B sample. For HAp-L, a clear inhibition of *E. coli* bacterial growth at 0.039 mg/mL was observed, but the MIC value was recorded at 0.02 mg/mL. The MIC value for *E. coli* bacterial growth was recorded at 0.078 mg/mL for the HAp-B sample. According to Tripathi [47], MBC is the concentration that leads to microbial death. In agreement with Tripathi, the MBC values at which the growth of *S. aureus* and *E. coli* was stopped by HAp-L were at concentrations of 1.25 mg/mL. On the other hand, Taylor et al. [48], in their studies on “determination of minimum bactericidal concentrations of oxacillin for *Staphylococcus aureus*: influence and significance of technical factors”, defined MBC as the smallest concentration that kills bacteria with a reduction of 99.9%. Pankey and Sabath [49], in their studies on “Clinical relevance of bacteriostatic versus bactericidal mechanisms of action in the treatment of Gram positive bacterial infections”, confirmed Taylor’s theory of MBC determination. In agreement with Taylor et al. [48] and Pankey and Sabath [49], the MBC value at which the development of *S. aureus* and *E coli* bacterial cells was stopped in the presence of HAp-L was 0.625 mg/mL. For MRSA, we could say that the growth of these bacterial cells was stopped at 2.5 mg/mL in the presence of HAp-L according to Taylor et al. [48] and Pankey and Sabath [49]. A slight decrease in the cellular viability of various Gram-positive bacteria (MRSA and *S. aureus*) and Gram-negative (*E. coli*) in the presence of HAp-B was evidenced without any reduction in cellular development by 99.9%.

Taking into account the fact that antibacterial agents that are considered bactericidal may only exhibit bacteriostatic activity in vitro [49] depending on the concentration at which the antibacterial materials were tested, the behavior of the HAp-B sample could be explained by the fact that the major component of the HAp-B samples was hydroxyapatite and the basil EO was poorly adsorbed onto the surface of hydroxyapatite nanoparticles.

Considering previous studies, the research presented in this paper contains the first study that highlights the MIC and MBC values of hydroxyapatite coated with lavender or basil EOs. The determination of MIC and MBC plays an important role in clinical applications when tracking bacterial response to antibiotic therapy [50,51]. In accordance with the research and controversy related to the bactericidal or bacteriostatic activity of materials with antibacterial properties, the studies presented in this work represent only a stage of our research on the determination of antimicrobial properties of materials based on hydroxyapatite coated with EOs (HAp-EOs). Current and future studies can provide useful information on the bacteriostatic or bactericidal activity of HAp-EOs on a wide range of bacterial strains in order to predict a favorable clinical outcome.

## 4. Conclusions

In this paper, the influence of the basil and lavender EOs on the morphological, physico-chemical and antibacterial properties of the HAp was analysed. The morphology of the HAp sample changed as particles agglomerated when coated with EOs. The FT-IR spectral investigations of the HAp-B and HAp-L samples revealed the presence of IR absorption bands specific to both HAp and EOs. This suggested that both basil and lavender EOs were incorporated into the HAp sample, which is in agreement with the SEM analysis and nitrogen adsorption-desorption. However, there was a reduced number of IR bands specific to basil EO present in the HAp-B sample in comparison with those specific to lavender EO identified in the HAp-L sample. The results obtained revealed that both HAp-B and HAp-L exhibited good antimicrobial activity. HAp-L and HAp-B were found to be effective in inhibiting the growth of *S. aureus*, *E coli* and MRSA bacterial strains. The results of this study suggested that the best antibacterial activity could be observed for the samples of hydroxyapatite coated with lavender EO. In conclusion, the determination of MIC and MBC values is relevant in clinical applications and allows a better understanding of the action of antibiotic agents. These results could significantly contribute to the development of new applications for the cosmetic and pharmaceutical industry. On the other hand, their use in bone reconstruction could help reduce the number of postoperative infections after various implants. Important future goals include the determination of the active antimicrobial components within the EO as well as the mechanisms involved in the process of inhibiting bacterial growth. The understanding of these mechanisms and the development of novel antimicrobial agents based on EOs available in nature presents tremendous potential for the discovery of alternatives to conventional antibiotics.

## Figures and Tables

**Table 1:** Specific surface area S_BET_, pore volume V_P_, mean pore diameter D_P_.

Sample	S_BET_ (m^2^/g)	V_P_ (cm^3^/g)	D_P_ (nm)
HAp	98.45	0.38	17.48
HAp-B	110.57	0.47	18.49
HAp-L	135.38	0.62	13.35

**Table 2:** The FTIR bands associated to the vibrational groups present in the HAp, HAp-B, HAp-L samples.

HApIR Band Wave Number	IR Band Assignment	HAp-B IR Band Wave Number	IR Band Assignment	HAp-L IR Band Wave Number	IR Band Assignment
470, 560, 600, 630	vibrations in [PO_4_]^3−^	470, 560, 600, 630	vibrations in [PO_4_]^3−^	470, 560, 600, 630	vibrations in [PO_4_]^3−^
875, 1420, 1450	vibrations in [CO_3_]^2−^	720	C–H deformations aromatic C-H out-of-plane bend	692	C–H deformations aromatic C–H out-of-plane bend
960, 1025, 1090	vibrations in [PO_4_]^3−^	875, 1420, 1450	C–O vibrations in [CO_3_]^2−^/C–H vibrations	835, 875, 1420, 1450	C–O vibrations in [CO_3_]^2−^/C–H vibrations
1645, 3338	O–H vibrations	960, 1025, 1090	vibrations in [PO_4_]^3−^	917	C–H deformations
		1640	H–-O–H vibrations	960, 1025, 1090	P–O vibrations in [PO_4_]^3−^
		3310	H_2_O vibrations	1235	C–O stretching of ester group
				1375	C–H in CH_3_ vibrations
				1640	C=C vibrations
				1740	C=O stretching vibrations
				2873, 2924	C–H vibrations in CH_3_ groups
				2972	C–H vibrations in CH_2_ group
				3400	O-H stretching vibrations

**Table 3:** Absorption intensities of lavender EO IR bands in lavender EO and HAp-L IR spectra.

Wavenumber (cm^−1^)	Absorption Intensities of Lavender EO IR Bands in Its IR Spectrum	Absorption Intensities of Lavender EO in HAp-L IR Spectrum
1235	0.01	0.024
1375	0.12	0.042
1420	0.058	0.042
1450	0.093	0.068
1740	0.11	0.064
2873	0.08	0.054
2924	0.139	0.077
2972	0.133	0.086
3400	0.046	0.04

**Table 4:** Inhibition zone diameters of HAp, HAp-B and HAp-L samples against MRSA, *S. aureus* 0364 and *E. coli* ATCC 25922 bacterial strains.

Bacterial Strain	Inhibition Zone (mm)		
	HAp	HAp-B	HAp-L
MRSA	-	9 ± 0.2	23.77 ± 0.3
*E. coli* ATCC 25922	-	14.65 ± 0.5	25.27 ± 0.7
*S. aureus* 0364	-	11.06 ± 0.4	25.83 ± 0.9
